# Anomalous Coronary Artery Variant of Common Origin from Right Coronary Cusp: A Case Report

**DOI:** 10.31729/jnma.4783

**Published:** 2020-04-30

**Authors:** Aziz Uliah, Ramesh Rana, Anish Hirachan

**Affiliations:** 1 Department of Radiology, Gautam Buddha Community Heart Hospital, Butwai, Nepal; 2 Department of Internal Medicine, Gautam Buddha Community Heart Hospital, Butwai, Nepal; 3 Department of Cardiology, Nepal Medicity Hospital, Laiitpur, Nepal

**Keywords:** *coronary angiography*, *coronary artery disease*, *coronary vessel anomalies*

## Abstract

Coronary artery anomalies are rare congenital variants of coronary artery anatomy accounting second most common cause of sudden cardiac death in young competitive athletes. A single ostium coronary artery anomalous is an extremely rare variant with an incidence of less than 0.004%. They may present as chest pain, arrhythmia, or sudden death. Recently, advanced imaging techniques such as computed tomography and magnetic resonance imaging coronary angiography are becoming the alternatives investigation for diagnosis. We reported a rare case of 50 years old lady who presented with acute chest pain with normal electrocardiography, echocardiography, and cardiac markers. Coronary Computed tomography angiography revealed anomalous coronary artery anatomy with both right and left coronary artery arising from the large common trunk of the right coronary cusp, left main coronary artery having trans-septal course, there was no flow-limiting coronary artery disease. She was medically managed with a single antiplatelet, beta-blocker, and statin therapy.

## INTRODUCTION

Coronary artery anomalies and variants are rare congenital disorders of coronary artery anatomy. It accounts for the second most common cause of sudden cardiac death in young competitive athletes.^1^ Anomalous of the coronary artery with a single ostium coronary artery is an extremely rare variant with an incidence of less than 0.004%.^2^ They may present as chest pain, arrhythmia, or sudden death. Recently, advanced imaging techniques such as computed tomography (CT) coronary angiography and magnetic resonance imaging coronary angiography are becoming the alternatives investigation for diagnosis.^3^

We report a rare case of 50 years old female diagnosed as an anomalous coronary artery with left and right coronary artery arising from a single common trunk-off the right coronary cusp.

## CASE REPORT

**Figure 1.**
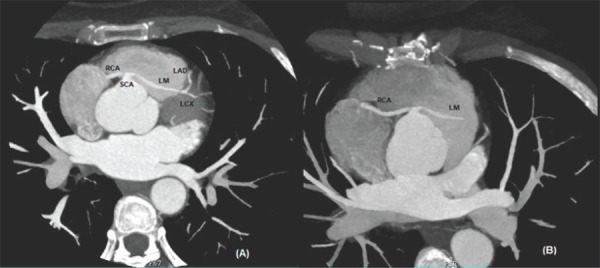
Axial images show a single coronary artery arising from right coronary sinus giving rise to right coronary artery (RCA) and left main (LM) arteries. (A) Left main passes through septal myocardium giving rise to Left Anterior Descending (LAD) and Left Circumflex (LCX). (B) RCA has a typical course within the right atrioventricular groove.

**Figure 2.**
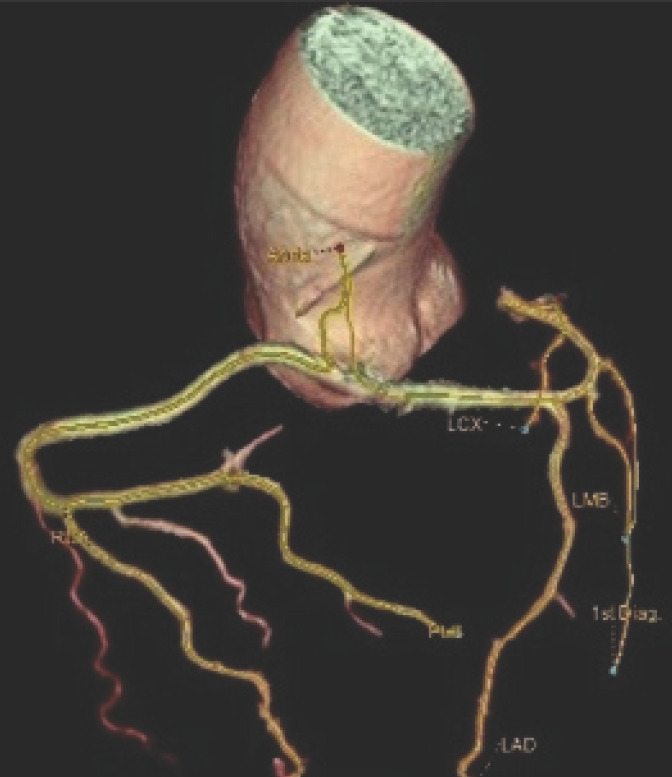
Tree VR images of anomalous origin of single coronary artery arising from right coronary sinus giving branches right coronary artery (RCA) and left main coronary artery (LM) which further divides into left anterior descending (LAD) and left circumflex (LCX) artery.

**Figure 3.**
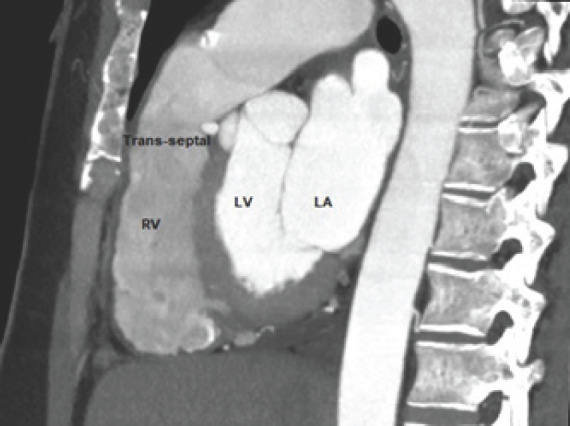
Oblique Sagittal reconstruction images of single coronary artery arising from the right coronary sinus beneath the level of the pulmonary valve. RV- right ventricle; LV- left ventricle; LA- left atrium.

We report a case of 50 years old female who visited the emergency department with a chief complaint of acute chest pain. She is a known case of hypothyroidism under thyroxine. However, she had no history of diabetes and hypertension. She is a non-smoker and non-alcoholic. On general physical examination, her vitals were stable with pulse rate 82 beats/ minute, blood pressure 100/80mmHg and systemic examination and laboratory investigations were within normal limit. Her ECG finding was normal; echo finding was normal left ventricular systolic and diastolic function with a normal left ventricular ejection fraction of 60%. Then, she was planned for CT coronary angiography in our center. It revealed anomalous coronary anatomy with both right and left coronary artery arising from large common trunk-off the right coronary cusp, left main coronary artery has a trans-septal course, there was no flow-limiting coronary artery disease (Figure 1, 2 and 3).

She was medically managed and discharged with single antiplatelet therapy, beta-blockers, and statin per-oral medications.

## DISCUSSION

Anomalous aortic origin of a coronary artery (AOCA) is a congenital abnormality of the origin arising from the sinus of Valsalva.^4^ Single ostium coronary artery is an extremely rare anomaly with a reported incidence of 0.04% of case of coronary angiography.^2,5^ Among different variants of anomalous coronary artery origins from the contralateral sinus of Valsalva, right-sided origin of left main coronary and left-sided RCA are the most common anomalies.^6^ For most variants of aberrant coronary origin, clinical significance and potential complications depend on its course. Depending upon the course of AOCA, the coronary arteries have 5 potential paths: pre-pulmonic, retro-aortic, intra-arterial, retro-cardiac, and trans-septal. In a trans-septal variant, the anomalous vessel passes through the septal muscle inferior to the plane of pulmonic valve giving off septal branches along the intra-myocardial course, whereas the anomalous vessel courses at the level of the pulmonic valve in the inter-arterial variant.^7^ Among various described courses of the coronary arteries, as they relate to the ascending aorta and pulmonary artery, the inter-arterial and trans-septal courses are clinically significant.

According to the coronary angiography done in 1,26,595 cases, the trans-septal variant was the most common in patients with the ectopic right-sided origin of the left coronary artery and right-sided single coronary types as reported in our case.^6^

It has been stated that the abnormal origin and course of anomalous coronary arteries could make them more prone to atherosclerosis. Occasionally, they may be associated with sudden death due to compression by the aorta and pulmonary artery. According to Villa et al, approximately 15% of such cases can develop myocardial ischemia without atherosclerosis as reported in our case,^7^ CT angiography revealed single coronary artery arising from the right coronary sinus and giving rise to right coronary artery (RCA) and left main artery (LMA), LMA passes through septal myocardium and giving rise to left anterior descending and left circumflex arteries, RCA had a typical course within the right atrioventricular groove. Nevertheless, there was no evidence of atherosclerotic or calcific plaque despite angina symptoms. The average age of symptomatic presentation was 35 years, the oldest reported of 83years old, in our case the patient became symptomatic at the age of 50 years old. Additionally, a stress test may assist in stratifying the risk of future cardiac ischemic events in anomaly patients.^7^

The use of CT angiography to define this anomaly gives scope for clinicians to perform long term follow up and possibilities to test anomalies functionally to judge hemodynamic significance. In this case, CT angiography not only diagnosed the anomaly of the coronary artery it also prevented the invasive method (conventional coronary angiography).

In conclusion, we report a rare anomaly of the common origin of right and left coronary from a single coronary ostium at the right sinus of Valsalva with a trans-septal course (malignant) course of the left coronary artery. These patients might be asymptomatic or may have events of sudden cardiac death. Patients are advised to avoid strenuous physical exertion.

## Consent:

**JNMA Case Report Consent Form** was signed by the patient and the original article is attached with the patient's chart.
